# Gelomyrtol for acute or chronic sinusitis: A protocol for systematic review and meta-analysis

**DOI:** 10.1097/MD.0000000000020611

**Published:** 2020-06-05

**Authors:** Yongcan Wu, Xiaomin Wang, Demei Huang, Caixia Pei, Shuiqin Li, Zhenxing Wang

**Affiliations:** a Department of Respiratory Medicine; b Department of Geriatrics; c Department of Gastroenterology, Hospital of Chengdu University of Traditional Chinese Medicine, Chengdu 610072, Sichuan Province, People's Republic of China.

**Keywords:** acute sinusitis, chronic sinusitis, gelomyrtol, protocol, systematic review

## Abstract

**Background:**

Sinusitis is a common condition worldwide, significantly affecting the quality of life of patients. Due to the limitations of conventional medicines, such as serious side effects and low efficacies, Gelomyrtol may be a promising treatment for sinusitis. As no related systematic review has been published, the purpose of this study will be to evaluate the safety and efficacy of Gelomyrtol for acute or chronic sinusitis.

**Methods:**

PubMed, EMBASE, the Cochrane Library, the Web of Science, the Chinese National Knowledge Infrastructure Database, the Chinese Biomedical Literature Database, the Wan Fang Database, and the Chongqing VIP Chinese Science, and Technology Periodical Database will be searched from their commencement until July 2020. Randomized controlled trials of Gelomyrtol for acute or chronic sinusitis will be selected in any language. Primary outcomes will include the Sino-Nasal Outcome Test-22 (SNOT-22) score, quality of life score as measured by SF-36, and the change in computed tomography (CT) score. Study selection, data extraction, and deviation risk assessment will be carried out by 2 investigators independently. RevMan V.5.3 software will be used to analyze the study data.

**Results:**

The study will provide high-quality evidence for estimating the efficacy and safety of Gelomyrtol in the treatment of acute or chronic sinusitis.

**Conclusions:**

This systematic review will explore whether Gelomyrtol is an effective and safe intervention in the treatment of acute or chronic sinusitis.

**Ethics and dissemination:**

As no patient data will be used in this study, ethical approval will not be required. The review will be published as an article or a conference presentation in a peer-reviewed journal.

**Registration:**

OSF registration number: DOI 10.17605/OSF.IO/MTEU2.

## 1 Introduction

Sinusitis, a disease that seriously affects the quality of human life, is mainly characterized by chronic inflammation of the nasal cavity and paranasal sinus mask. The incidence of sinusitis is higher in women than in men, and children younger than 15 years and adults between 25 and 64 years are affected the most.^[1]^ Sinusitis can be classified into 4 categories according to the duration of the symptoms as follows: acute sinusitis (symptoms last <4 weeks), subacute sinusitis (symptoms last about 4–12 weeks), chronic sinusitis (symptoms last >12 weeks), and recurrent sinusitis (≥4 acute episodes occur in any year lasting at least 7 days).^[2]^ One National Health Interview Survey found that up to 14.7% of individuals reported suffering from sinusitis in the past year.^[3]^ Sinusitis is also responsible for enormous direct and indirect medical costs that affect the global economy.^[4]^ At present, the widely accepted and recommended medical treatments for acute or chronic sinusitis are local administration of nasal steroids, nasal irrigation, antibiotic treatment, or systemic administration of steroids. Endonasal sinus surgery is recommended for patients who cannot benefit from drug treatment.^[5,6]^

Although there are many therapeutic measures to improve sinusitis, management is often challenging. Sinusitis is common in the general population, and it can affect the quality of life more than angina or respiratory disease.^[7]^ Studies have shown that sinusitis causes 57% of patients to be absent from work, and 28% of patients to experience anxiety and depression.^[8,9]^ Of the currently available sinusitis medications, the risks of using corticosteroids far exceed the benefits,^[10]^ and the International Consensus Statement on Allergy and Rhinology (ICAR) guidelines note that there is insufficient evidence for antibiotic use.^[11]^ Therefore, there is no consensus on the optimal management strategy.

Gelomyrtol, a phytomedicine, is extracted from the leaves of Myrtaceae. It has been shown to have secretolytic, mucolytic, secretomotoric, and anti-inflammatory properties.^[12–15]^ The efficacy of Gelomyrtol in the treatment of acute or chronic sinusitis has been confirmed in randomized controlled trials (RCTs) and recommended in German and international treatment guidelines.^[16,17]^ Therefore, Gelomyrtol has the potential to be an effective adjuvant therapy for acute or chronic sinusitis. However, there is little evidence of its curative efficacy for acute or chronic sinusitis. Therefore, the aim of this study is to provide a comprehensive systematic review on the safety and efficacy of Gelomyrtol for patients with acute or chronic sinusitis.

### 1.1 Review objectives

We aim to provide a comprehensive systematic review and meta-analysis of the safety and efficacy of Gelomyrtol for patients with acute or chronic sinusitis. The further purpose is to explore the potential regulatory factors of intervention effects and the sources of heterogeneity among studies, such as the characteristics of specific interventions, the tested population or the study design.

## 2 Methods

### 2.1 Protocol and registration

This study was registered on OSF. Registration number: DOI 10.17605/OSF.IO/MTEU2. The protocol was designed according to the Preferred Reporting Items for Systematic Reviews and Meta-Analyses Guidelines for Protocols (PRISMA-P).^[18]^

### 2.2 Criteria for including studies in the review

#### 2.2.1 Types of studies

The study will include parallel group, RCTs that were reported in any language. Surgical intervention trials, adverse drug reaction (ADR)-related studies, mechanistic trials, case reports, and experimental studies will be excluded to minimize biased estimates of the efficacy of the treatment.

#### 2.2.2 Types of participants

Participants who meet the diagnostic criteria of acute or chronic sinusitis will be included.^[2,19,20]^ The study will include participants of any age, any race, and both sexes.

#### 2.2.3 Types of interventions

##### 2.2.3.1 Experimental interventions

Gelomyrtol for acute or chronic sinusitis regardless of the dose, administration route, frequency, and administration regimen (either alone or in combination with another drug) will be included.

##### 2.2.3.2 Comparator interventions

Placebo, western medicine treatment, or non-drug treatment (regardless of the dose, administration route, frequency, and administration regimen) will be included. Studies of all groups containing Gelomyrtol will be excluded.

#### 2.2.4 Types of outcome measures

##### 2.2.4.1 Primary outcomes

1. Score on the Sino-Nasal Outcome Test-22 (SNOT-22);
2. Quality of life as measured by SF-36; and
3. Change in computed tomography (CT) score.

##### 2.2.4.2 Secondary outcomes

1. Total nasal symptom score;
2. Total effective rate;
3. Recurrence rate;
4. Treatment-related adverse events; and
5. Objective physiological measures: nasal peak flow, nasal volume, nasal cross-sectional area, nasal nitric oxide, and ciliary function (including saccharine clearance time).

### 2.3 Search methods for the identification of studies

#### 2.3.1 Electronic searches

PubMed/MEDLINE, EMBASE, the Cochrane Library, the Web of Science, the Chinese National Knowledge Infrastructure (CNKI) Database, the Chinese Biomedical Literature Database (CBM), the Wan Fang Database, and the Chongqing VIP Chinese Science and Technology Periodical Database (VIP) will be comprehensively searched from their commencement until July 2020. We will include all relevant RCTs that examine the effectiveness of Gelomyrtol for acute or chronic sinusitis. There will be no language restrictions for the included RCTs. Searches will be carried out by 2 investigators (PCX and HDM), according to the syntax of each database and the principles of Population, Intervention, Comparison, Outcomes, and Study design (PICOS) in evidence-based medicine. The retrieval strategy will be developed and provided upon commencement of the study. For example, search terms in PubMed will contain: ∗sinusitis (using ∗ as an unlimited truncation strategy to capture all variations of sinusitis), “Gelomyrtol," “Myrtol," “Myrtol standard," “ELOM-080," and “Randomized." The terms will be used in all possible combinations to yield the maximum number of studies. The example of specific search for PubMed is shown in Table 1.

**Table 1:**
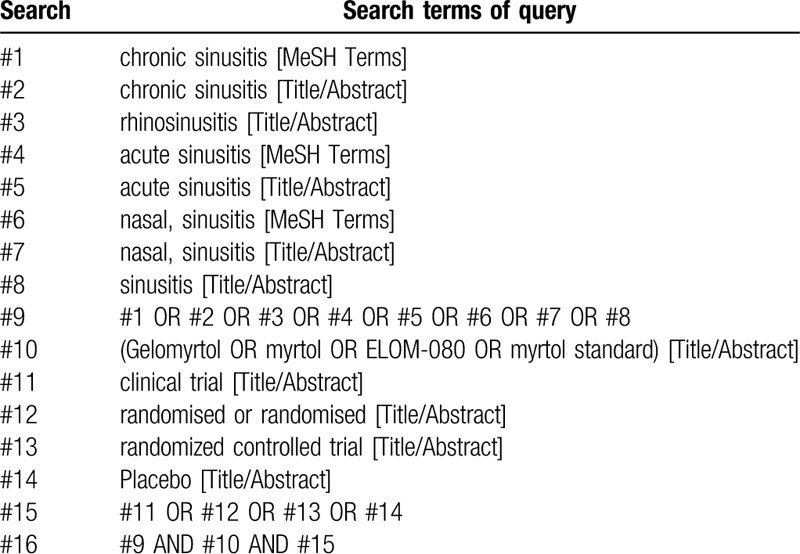
PubMed search strategies.

#### 2.3.2 Searching other resources

We will retrieve unpublished data from ongoing studies in the NIH clinical registry Clinical Trials.Gov (https://www.clinicaltrials.gov/), the International Clinical Trials Registry Platform (ICTRP) (https://www.who.int/ictrp/en/), the Australian New Zealand Clinical Trials Registry (https://www.anzctr.org.au/), and the Chinese Clinical Registry (http://www.chictr.org.cn/index.aspx). Other publications, such as relevant systematic reviews and meta-analyses, will be reviewed to identify additional trials.

### 2.4 Data collection and analysis

#### 2.4.1 Selection of studies

**Figure 1:**
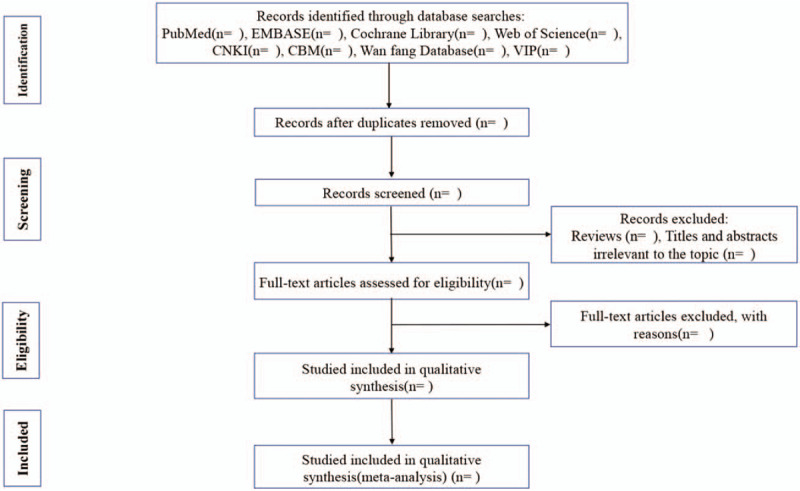
Flow diagram of the study selection process.

We will import the search results into Endnote X (V.9.0). Two investigators (PXC and HDM) will evaluate the retrieved studies based on the inclusion criteria. They will conduct a preliminary screening of the title and abstract to exclude articles that do not meet the requirements. Unmatched studies will be moved to the trash box in Endnote X. The reasons for the exclusion of selected studies will be recorded in an Excel table. The next step will be to read the full text and to evaluate the reason for inclusion based on the full text. Two investigators will examine the reference list to confirm possible missing studies. The results of the selection process will be cross-checked by 2 investigators. Any differences will be resolved through discussion and a consensus. A third investigator (LSQ) will arbitrate unresolved arguments. Each eligible study will be assigned a research ID using the following format: first author's last name + space + year of publication (eg, Wang 2019). The selection of studies is summarized in a PRISMA flow diagram (Fig. 1).

#### 2.4.2 Data extraction and management

Two investigators (WXM and WYC) will review the eligibility of each included study and extract data by using a standardized template. This standardized template will include the following information: article information (title, list of authors, year of publication, sample size, design, country, sponsor), participant information (age, sex, number of samples in the group, acute or chronic sinusitis diagnostic criteria), intervention details (intervention therapy, comparator therapy, dosage, duration, administration route, co-intervention), outcome details (measuring methods, adverse events). Any differences identified during data cross-checking will be resolved through discussion and the recommendation of a third investigator (LSQ).

#### 2.4.3 Assessment of bias risk of the included studies

The bias risk of each included study will be assessed by using Cochrane Collaboration's tools in randomized trials.^[21]^ Two investigators (WXM and WYC) will input the relevant information of each study into RevMan software.^[22]^ The quality of the methodology of each included study will be evaluated from 7 perspectives, including random sequence generation, allocation concealment, blinding of participants and personnel, blinding of outcome assessment, incomplete outcome data, selective reporting, and other potential sources of bias.^[23]^ The investigators will assess the risk of the 7 biased projects one by one, and grade the risk of each item into 1 of 3 levels as follows: high, low, or unclear. Differences will be settled through discussion. A third investigator (LSQ) will be consulted, if necessary, to reach a consensus. If the essential information is lost or unreported, the authors of the original study will be contacted for the risk of deviation assessment. The assessment results will be displayed in the bias risk summary chart.^[21]^

#### 2.4.4 Measures of treatment effect

Risk ratios with 95% confidence intervals (CIs) will be used to evaluate dichotomous outcomes. Continuous outcomes will be calculated by using the mean difference or standardized mean difference (SMD) with 95% CIs. The MD will be applied if the same scale is used to estimate an outcome in different studies. The SMD will be applied for different measurement tools.

#### 2.4.5 Unit of analysis issues

Before statistical analysis, the units of the outcomes from the different studies will be transformed into the International System of Units.

#### 2.4.6 Dealing with missing data

Data may be missing from studies whether intentionally or unintentionally. We will contact the corresponding author of such studies for this reason. After the investigators confirm that the data were missing, the available data will be analyzed.^[22]^ The missing data will not be used in the preliminary analysis. However, the impact of the missing data will be assessed in the sensitivity analysis. The potential impact of the missing data on the final result of the review will be analyzed in the discussion.

#### 2.4.7 Assessment of heterogeneity

We will use *χ*^2^ tests to evaluate the statistical heterogeneity in the forest plot by RevMan V.5.3. According to the Cochrane Handbook, *P* values <0.1 will be identified as significant.^[23]^ Furthermore, *I*^2^ values will be analyzed to quantify the influence of the statistical heterogeneity. Heterogeneity will be classified into 4 categories according to the Cochrane Handbook as follows: 0% to 40%, might not be important; 30% to 60%, indicates moderate heterogeneity; 50% to 90%, represents substantial heterogeneity; and 75% to 100%, suggests considerable heterogeneity.

#### 2.4.8 Assessment of reporting biases

We will use a funnel plot to assess the reporting bias if ten or more studies are included.^[23]^

### 2.5 Data synthesis

The data of clinical studies will be input into Revman software (V.5.3) for data synthesis. The data will be synthesized and analyzed according to the level of statistical heterogeneity. If the study is homogeneous enough in terms of design and comparison, we will use a random-effect model for meta-analysis. If there is a large heterogeneity in the included trials, meta-analysis will not be carried out. We will try to find out the root causes of heterogeneity from various aspects and provide a narrative and qualitative summary.

#### 2.5.1 Subgroup analysis and investigation of heterogeneity

If there is significant heterogeneity in the included trials, then we will conduct subgroup analysis according to the region of the studies, age, stage of the subjects, types of treatments, and different outcomes.

#### 2.5.2 Sensitivity analysis

Sensitivity analysis will be used to identify the robustness of the review conclusions, such as methodological weaknesses, missing data, and heterogeneity qualities.

#### 2.5.3 Evaluation of the quality of evidence

Online Grading of Recommendations Assessment, Development and Evaluation (GRADE) will be used to independently assess the quality of evidence for each outcome by the reviewers.^[24]^ The evidence level will be classified into 4 possible ratings as follows: very low, low, moderate, or high.

## 3 Discussion

Gelomyrtol has been proved to have the effects of anti-inflammation, dilution of mucus, and so on. Its curative effect has been confirmed. However, the efficacy and safety of Gelomyrtol in the treatment of acute or chronic sinusitis are not clear. Therefore, we believe that the results of this meta-analysis will provide some reference for clinicians and researchers.

### 3.1 Amendments

If there are any amendments in this protocol, we will give the date of each amendment, describe the situation of the change, and give the reason.

## Acknowledgments

The authors thank TopEdit (www.topeditsci.com) for its linguistic assistance during the preparation of this manuscript.

## Author contributions

**Methodology:** WZX and WYC

**Resources:** WYC and WXM

**Software:** PCX and HDM

**Supervision:** LSQ

**Writing – original draft:** WYC and WXM

**Writing – review & editing:** WZX and LSQ
